# Management of severe trauma worldwide: implementation of trauma systems in emerging countries: China, Russia and South Africa

**DOI:** 10.1186/s13054-021-03681-8

**Published:** 2021-08-09

**Authors:** Jing Zhou, Tianbing Wang, Igor Belenkiy, Timothy Craig Hardcastle, Jean-Jacques Rouby, Baoguo Jiang, for the International Trauma Rescue & Treatment Association (ITRTA) Study Group, Demetrios Demetriades, Hans J. Oestern, Hiroaki Iwase, Mao Zhang, Pierre Bouzat, Timothy J. Coats, Tobias Gauss, Youzhong An

**Affiliations:** 1 National Center for Trauma Medicine, Trauma Center, Department of Orthopedics and Traumatology, Peking University People’s Hospital, Beijing, China; 2 Department of the Trauma and Orthopedics, Pavlov First Saint-Petersburg State Medical University, St. Petersburg, Russia; 3 Trauma and Burns Service, Inkosi Albert Luthuli Central Hospital, Durban, South Africa; 4 Department of Surgery, Nelson R Mandela School of Clinical Medicine, UKZN, Durban, South Africa; 5 Multidisciplinary Intensive Care Unit, Department of Anesthesiology and Critical Care Medicine, Sorbonne University, La Pitié-Salpêtrière Hospital, Assistance-Publique-Hôpitaux-de-Paris, Paris, France; 6 Department of Trauma and Orthopedics , St. Petersburg I. I. Dzhanelidze Research Institute of Emergency Medicine, St. Petersburg, Russia

**Keywords:** Trauma system, Trauma care, Trauma center, Pre-hospital care, In-hospital care, Trauma registry, Chinese trauma system, Russian trauma system, South African trauma system

## Abstract

As emerging countries, China, Russia, and South Africa are establishing and/or improving their trauma systems. China has recently established a trauma system named “the Chinese Regional Trauma Care System” and covered over 200 million populations. It includes paramedic-staffed pre-hospital care, in-hospital care in certified trauma centers, trauma registry, quality assurance, continuous improvement and ongoing coverage of the entire Chinese territory. The Russian trauma system was formed in the first decade of the twenty-first century. Pre-hospital care is region-based, with a regional coordination center that determines which team will go to the scene and the nearest hospital where the victim should be transported. Physician-staffed ambulances are organized according to three levels of trauma severity corresponding to three levels of trauma centers where in-hospital care is managed by a trauma team. No national trauma registry exists in Russia. Improvements to the Russian trauma system have been scheduled. There is no unified trauma system in South Africa, and trauma care is organized by public and private emergency medical service in each province. During the pre-hospital care, paramedics provide basic or advanced life support services and transport the patients to the nearest hospital because of the limited number of trauma centers. In-hospital care is inclusive with a limited number of accredited trauma centers. In-hospital care is managed by emergency medicine with multidisciplinary care by the various specialties. There is no national trauma registry in South Africa. The South African trauma system is facing multiple challenges. An increase in financial support, training for primary emergency trauma care, and coordination of private sector, need to be planned.

**Supplementary Information:**

The online version contains supplementary material available at 10.1186/s13054-021-03681-8.

## Introduction

Trauma is a worldwide public health issue with economic implications. Between 1980 and 2017 road injury was the sixth cause of death in 195 countries and territories [1]. Numerous studies have demonstrated that trauma systems improve trauma outcomes [2–11]. A review of trauma systems across the world can identify differences and challenges, determine or adjust policy and promote improvement measures.

This article describes trauma systems in emerging countries, China, Russia, and South Africa.

## The Chinese trauma system

**Fig. 1:**
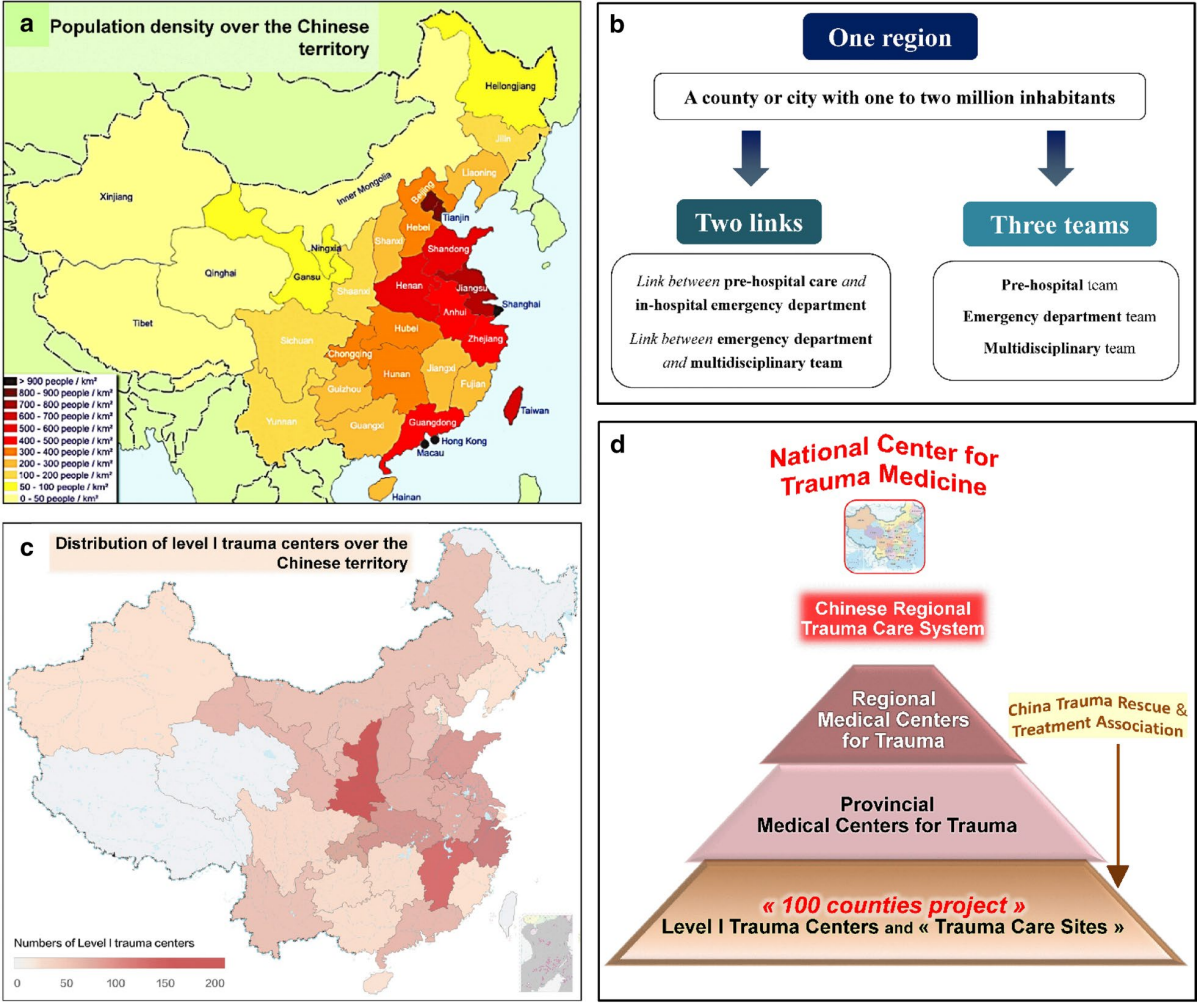
The Chinese Regional Trauma Care System “One region, Two links, Three teams” **a** Map of China showing the heterogeneous distribution of the population density over the national territory **b** Characteristics of the Chinese Regional Trauma Care System. The basic unit is a county or a small city including one to two million inhabitants with a level I trauma center in an existing large-scale hospital. The Chinese Regional Trauma Care System provides pre-hospital care from the emergency call center, the emergency care on scene and the transportation, and in-hospital care, which is provided either in a large-scale hospital (served as “level I trauma center”) with the availability of a multidisciplinary team or in five to six small hospitals (served as “trauma care sites”) with the ability to provide basic trauma care. Transfer between trauma care sites and level I trauma centers is possible according to pre-determined triage algorithm, creating “regional close-loop trauma care”. **c** Map of China showing that the number of level I trauma centers over the national territory in relation to the population density. **d** In order to balance the development of different regions, take into consideration regional specificities and integrate the trauma centers hierarchically nationwide, the government is proposing a national plan whose implementation will be driven by the National Center for Trauma Medicine. In large regions composed of several provinces, a Regional Medical Center for Trauma and several Provincial Medical Centers for Trauma will be established. Regional level I Trauma Centers which form the basic network of the Chinese Regional Trauma Care System have been mostly established (Fig. 1c) under the direction of the CTRTA

In 2018, China had a population of approximately 1.4 billion inhabitants heterogeneously spread across 9.6 million km^2^ (Fig. 1a) [12]. Mortality from trauma is around 30/100,000 in urban areas and approaches 50/100,000 in rural areas. It is the fifth cause of nationwide mortality.

### The Chinese trauma system: territorial organization and pre-hospital care

In the early 2000s, the huge expansion of highway networks and vehicles in China was associated with a marked increase in trauma injuries. The inadequate existing emergency rescue system (see Additional file 1 Historical background) resulted in a high mortality rate [13, 14]. The China Trauma Rescue & Treatment Association (CTRTA) and National Center for Trauma Medicine (NCTM) were founded in 2016 and 2019 to construct a trauma system adapted to Chinese specificities.

In 2016, the “Chinese Regional Trauma Care System” (CRTCS) was created under the government’s auspices, and the first step called “100 Counties Project” was implemented (Fig. 1b–d). In 2020, approximately 1000 level I trauma centers had been created in existing hospitals of more than 700 counties, covering 28 out of the 34 provinces and including over 200 million inhabitants.

Each county or city has an emergency call center which receives the calls on the national emergency number “120” and dispatches paramedic-staffed ambulances Transportation to the appropriate trauma center is decided according to an established algorithm. There is an information system providing patients’ clinical information to the receiving facility. Currently there is no public pre-hospital air transportation.

### In-hospital care

The in-hospital care is “inclusive” as every trauma patient irrespective the injury severity is included within trauma centers in existing hospitals. Level I trauma centers are located in multidisciplinary large-scale hospitals whereas level II trauma centers (“trauma care site”) are located in smaller hospitals. In most trauma centers, there is no specialized “trauma surgery”. The in-hospital trauma care leadership can be assumed by orthopedic and general surgeons, emergency physicians, or other specialists. In level I trauma centers, multidisciplinary teams are available for trauma care management. The vast majority of Chinese provinces are now equipped with level I trauma centers and there is a government national plan to cover the whole territory (Fig. 1c, d).

### Trauma system governance, trauma registry and performance improvement

In 2016, the CTRTA created a national trauma registry and databank. Level I trauma centers of the CRTCS are required to record clinical data of trauma patients. In 2021, more than 700,000 trauma patients had been registered. The management of the CRTCS is mainly led by the CTRTA and NCTM under the policy and auspices of the government. The CTRTA and NCTM have established standards for trauma care quality, including regular trauma training courses and meetings required for the certification of trauma centers every 3 years. The level I trauma centers are required to establish the quality control committee. The regular trauma meetings are required for reviewing the process of trauma care. The CTRTA will make regular review of trauma data for monitoring and improving the outcomes of trauma care.

### The Chinese trauma system facing major disasters

China has experienced several high-magnitude earthquakes during the twenty-first century such as the 2008 Wenchuan Earthquake and 2010 Yushu Earthquake that were associated with mass casualties. At those times no definitive trauma systems existed (see Additional file 1 Historical background). With national support, the central government mobilized national resources to provide efficient rescue to victims [15, 16], while the regional government facilitated the access to regional medical facilities. Despite the successful rescue, many deficiencies requiring improvements were identified [17, 18].

During the Wenchuan Earthquake, the appropriate assessment of needs and transport logistics, markedly affected by destruction of communication routes, were key issues for trauma patients’ outcomes [19]. For the severely injured patients, immediate emergency care on the scene was critical, justifying the rapid implementation of mobile medico-surgical hospitals on site [20]. To optimize the medical response and fit the degree of emergency, a triage protocol was implemented [21]. Since many physicians without qualifications in trauma care were sent to Sichuan, general training in basic trauma care appeared essential to face future disasters.

### Current challenges for the Chinese regional trauma care system

The CRTCS has gained rapid coverage and trauma care has been improved. The mortality of trauma had decreased slightly from 2016 to 2019, especially in urban areas. [22–24]

Further improvements still need to be undertaken. Concerning the pre-hospital care, aeromedical transportation needs to be developed in order to optimize neurotrauma care and rescue of severely injured patients in regions with difficult access. With the establishment of air transportation, the development of infrastructures, equipment, aeromedical care protocols, staff training and certification will have to be carried out. The creation of a “trauma surgery” specialty is indispensable to improve in-hospital care. The establishment of protocols for massive transfusion, resuscitative endovascular balloon occlusion of the aorta, and the creation of hybrid emergency rooms will be considered.

The CRTCS, initially centered on densely populated counties and cities, needs to be extended to sparsely populated border and rural areas. It will have also to face the demographic evolution of China with an increasing aging population.

## The Russian trauma system

Russia covers an area of 17,125,191 km^2^ with 146,748,590 residents at 2020. The population is concentrated in the triangle St. Petersburg-Sochi-Irkutsk. In 2018, the number of injuries was 12,930,453 (89 per 1000 population) [25], 12.4 people per 100,000 populations died from road crash injuries [26], and the total number of ambulance visits for trauma 3,800,537 (25.9 per 1000 population) [27].

### The Russian trauma system: territorial organization and pre-hospital care

**Fig. 2:**
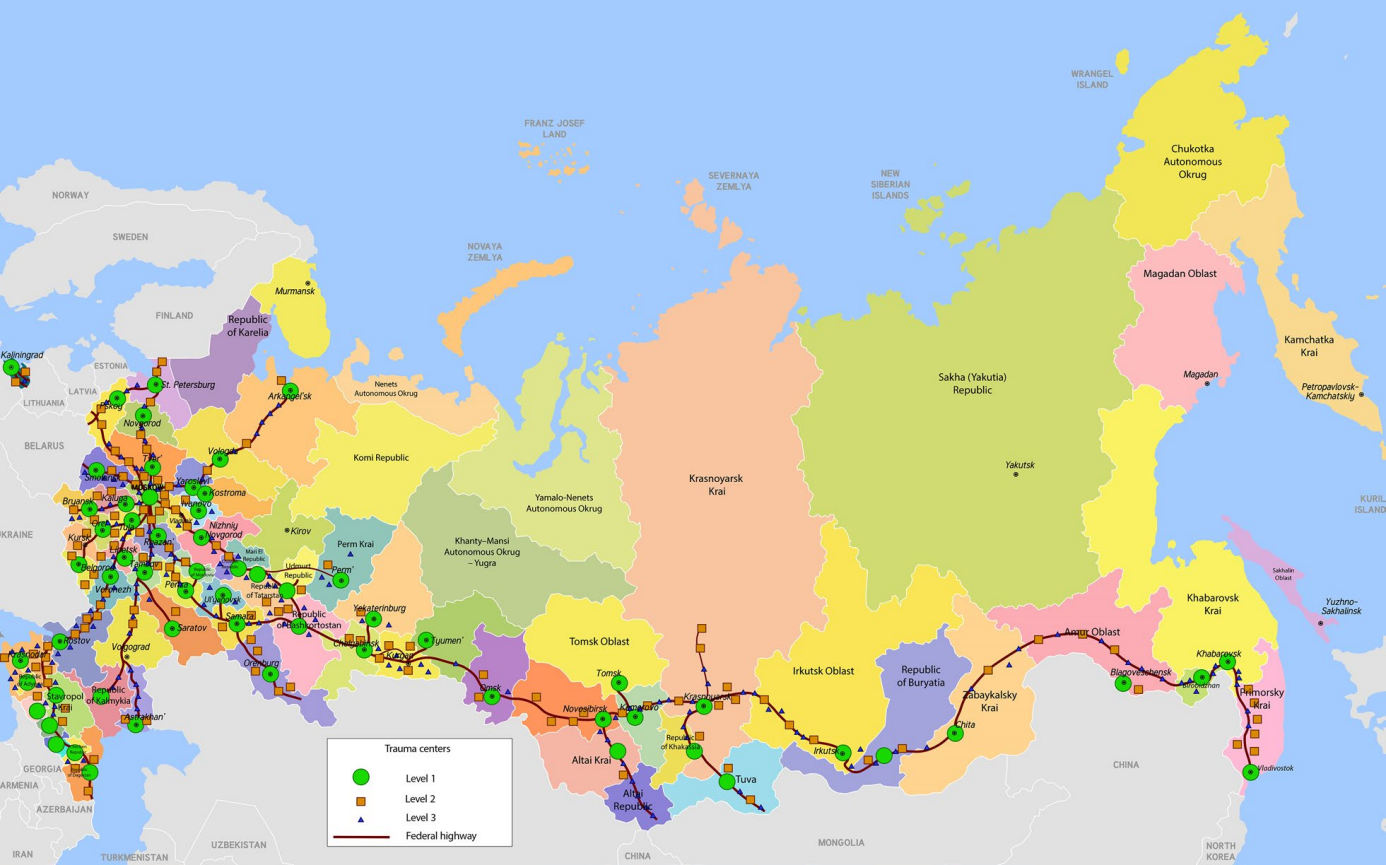
Location of trauma centers in Russia. There are 1500 trauma centers distributed over the Russian territory, 586 of which are level I and II centers. They are located in large cities and along the “trauma axes” (trunk roads). The exceptions are large areas with low population density in the north and north-east of Russia. There is a wide network of outpatient clinics with 24 h trauma and general surgeons on duty. Patients with minor isolated injuries come to these clinics themselves. Further they can be sent home after treatment or transported to the hospitals depending on nature of injury. Patients with moderate and severe injuries are transported to the nearest trauma centers by ambulances. Taking into account that most severe injuries occur on the roads, all new cars and trucks have been equipped with satellite emergency systems since 2019. This can significantly reduce arrival time of the ambulances to the place of accident

The modern trauma system was formed in the first decade of the twenty-first century (see Additional file 2 Historical background) [28] with a distribution of trauma centers adapted to the regional population (Fig. 2).

In each region, medical call centers answer the national emergency number “112”. The recommended time for ambulance arrival at the scene ≤ 20 min is not always met. In the Arkhangelsk region (north of Russia), the ambulance team arrives at the scene in 20 min only in 3.3% of cases, and the “golden hour” included only 74.4% of accidents [29]. There are three classes of ambulances: class A is for non-emergency use; class B is physician-staffed for medical emergencies; class C is physician-staffed for emergency resuscitation and multiple trauma. Recommendations and guidelines for pre-hospital care are not unified and vary from region to region. Each regional coordination center determines which team goes to the scene and the nearest hospital where the victim is transported. The most advanced system based on a special software package is available in the Moscow region [30] and in Saint-Petersburg [31].

### In-hospital care

Russian trauma centers are located in multidisciplinary hospitals and divided into three levels. Patients with severe injuries are transported to level I and II trauma centers equipped to treat severely injured patients. Medical teams 24 h/day on duty consist of a trauma surgeon, a neurosurgeon, an intensive care physician, with the surgeon most commonly as team-leader.

### Trauma system governance, trauma registry and performance improvement

The Russian trauma system is directed and managed by the Ministry of Health. There is no national trauma registry. Each region collects information on the number of injuries according to the International Classification of Diseases codes and sends it to the Ministry of Health. Based on these data, decisions about improvement of trauma systems are made on the Russian Federation State level [32]. Improvements relate to the amount of funding, the type of patient transportation, the identification of weakness and the determination of educational needs.

### The Russian trauma system facing major disasters and terrorist attacks

In the first 15 years of the twenty-first century, 879 people were victims of terrorist attacks in Russia, and more than 2300 were injured [33]. The most significant was the hostage-taking at the Beslan school on 2004, which involved more than 1000 hostages, mostly children and killed more than 300 people [33]. It started on September 1, the first day of the school year, and ended on September 3 by terrorist auto-explosions followed by the rescue operation. During the 48 h, 31 specialized ambulances were routed to the scene, and an airmobile hospital was deployed by the All Russian Center of Disaster Medicine. After the explosion, medical assistance was immediately provided to injured children, using the methods of military surgery and triage. One hundred and forty-six children were evacuated to hospitals in Moscow and Rostov. Ten children died. Effectiveness of medical care and evacuation was provided through integration of all the medical services of the Ministry of Health, the Ministry for Civil Defence, and the Ministry of Defence [33].

Analysis of the 12,836 articles on terrorism contained in the database of the Russian Science Citation Index revealed that only 1.5% concerned medical aspects. The same analysis of the 18,334 articles on terrorism contained in the Scopus database revealed 22.7% of medical publications. This analysis outlined the weakness of Russian medical publications on terrorism and called for a better coordination between the three ministries [34].

### Current challenges for the Russian trauma system

The development of aeromedical care is a prerequisite for the efficiency of the Russian health care system, owing to its specific climatic and demographic conditions. It is important both for remote areas and megacities. In Russia, the existing aeromedical service was initially carried out only by the Ministry for Civil Defence, Emergencies and Elimination of Consequences of Natural Disasters. The recent development of aeromedical services was initiated by the Ministry of Public Health and the Ministry of Industry and Trade under the instructions of the President of the Russian Federation.

Joint Stock Company National Air Ambulance Service, an operator designed to provide emergency aeromedical care throughout Russia, was founded in 2017. The project to create the National Air Ambulance Service was initiated by Rostec State Corporation at the end of 2018. In 2019, 49 regions were involved, and in 2020 their number increased to 70. The cost is funded by the Obligate Medical Insurance fund, and additional resources for payment are issued from the budgets of the Russian Federation. Aviation evacuations from the scene to medical facilities represented 30.9% of cases in 2019 and inter-hospital aeromedical transportations accounted for 68.9%. The lack of a single system and organization that coordinates interaction between Ministries, air services and hospitals in each region, explains that existing aeromedical services are often not used or insufficiently used [35]. The new unified National Air Ambulance Service should correct these deficiencies.

It is planned to implement new blocks of emergency care with separation in “red” zone for severe patients, “yellow” for patients of moderate severity and “green” for patients with isolated injuries. The fleet of ambulance cars is also being updated, and aeromedical helicopter numbers are increasing. The government has set targets to be achieved by the end of 2020. A reduction in mortality from road traffic injuries to 10 per 100,000 populations, was achieved by 2019. The next targets are bringing 80% of patients with polytrauma to level I and II trauma centers, and reducing the transportation time to less than 1 h.

## The South African trauma system

South Africa has 58 million people spread across 1,221,037 km^2^ and nine provinces. Provincial health reports to a National Department in the public sphere caring for 84% of the population [36, 37]. A fee-for-service private sector covers the rest, with inequality of care across the country [38]. Trauma cases per year are 12/1000 population with about 50,000 mortalities divided approximately 60:40 blunt to penetrating trauma [39, 40]. Trauma has a lower priority resulting from government’s primary health care focus.

### The South African trauma system: territorial organization and pre-hospital care

Health care is enshrined in the 2003 National Health Act, which requires provincial oversight of the health facilities, services and human resources in the nine provinces. Independently private hospital groups and Emergency Medical Services (EMS) have their own footprint [41]. There is no one unified trauma system: each province has both public and private EMS that cooperate and coordinate to varying degrees. Each system is at differing states of formalization. The first “trauma center” was established in 1962 and EMS became established in the late 1970’s with “paramedics” similar to the American system (see Additional file 3 Historical background).

**Fig. 3:**
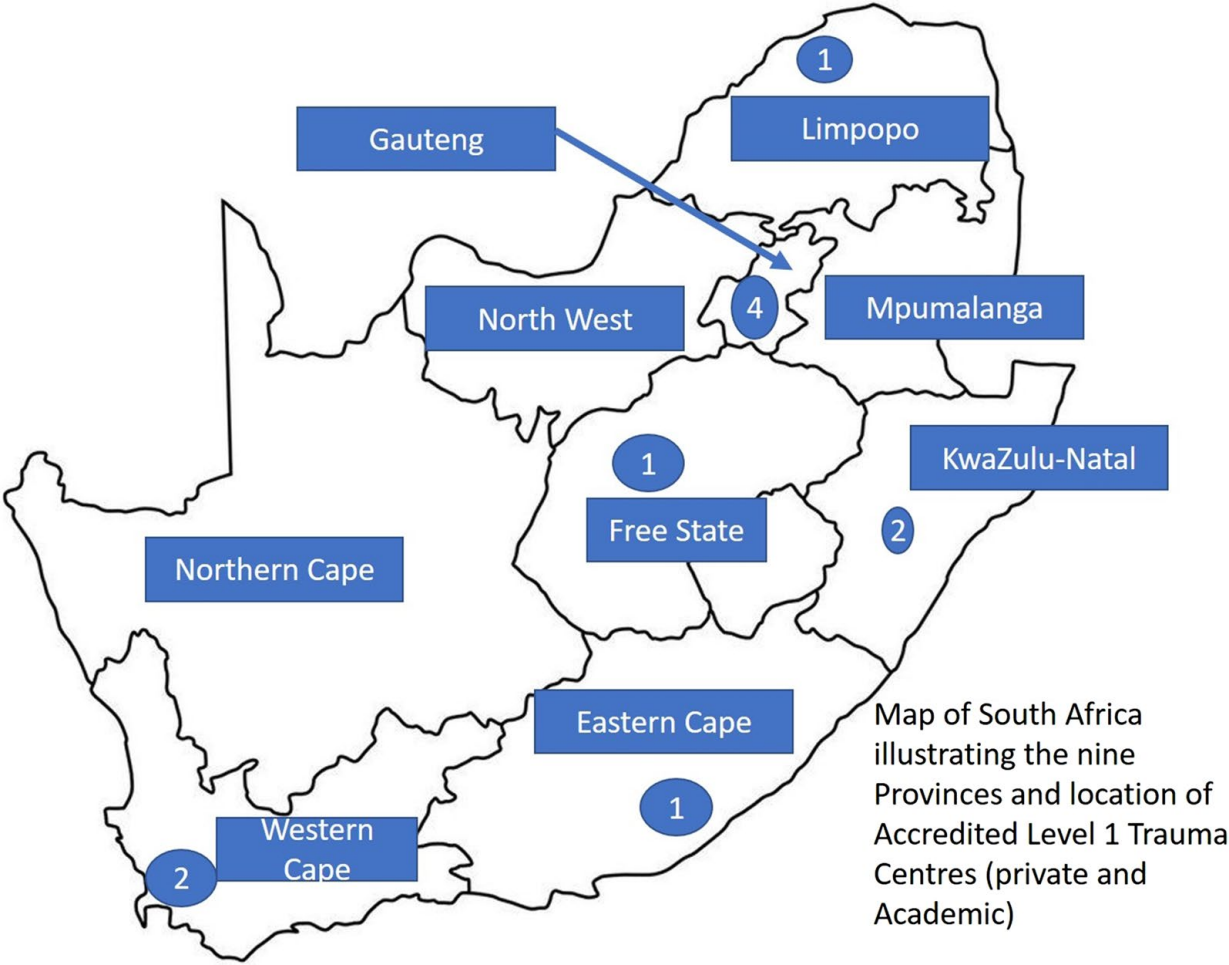
Trauma facilities in the nine provinces of South Africa. Six provinces have one or several trauma centers. With limited numbers of trauma centers, most patients do not go directly to a major facility and require subsequent transfer delaying definitive care. The government “primary health care” approaches are somewhat restrictive. Private services will often take the patient to a more appropriate facility due to cost-drivers. The country is well covered by helicopter Emergency Medical Service: four private companies (Netcare, ER24, HALO-aviation, LenMed) and one non-profit company, the Red-Cross AMS which operates in Cape Town, Oudtshoorn and Kwazulu-Natal. All are equipped for advanced life support and difficult access (mountain or sea rescue and high-rise rescue). Staffing is either doctor-paramedic or dual paramedic emergency care practitioner level

Since 1985 there is a national EMS telephone number (currently the 112) that routes to the nearest provincial EMS control center, allowing for coordinated dispatch of public paramedic-staffed ambulances and rescue services [42]. This does not apply to the private sector, with unlinked call-centers, despite the legislated requirement to update the public coordinating centers. Paramedics’ training is based on 4-year degree programs (see Additional file 3 Historical background). From dispatch of basic or advanced life support (ALS) services and Fire & Rescue, the nationally mandated response time is under 15 min for 90% of urban calls and extended to 45 min for rural areas [43]. Rapid transfer follows essential treatment on scene to the nearest hospital in most cases because of the limited number of trauma centers (Fig. 3).

South African aeromedical transportation combines private and public services. EMS use field triage which success is limited by major shortage of ALS providers, and patient flow protocols in public sector services [44]. At the state sector level and for certain of the larger nationally active private services (Netcare911 and ER24) communication systems and command centers linking the on-scene practitioner with the receiving hospital exist, mandated by various laws.

### In-hospital care

There are no trauma-only hospitals. Trauma Centers are accredited by the Trauma Society of South Africa (TSSA) [45, 46]. Trauma is initially managed by emergency medicine with multidisciplinary definitive care by the various specialties. Trauma surgery is present mainly in the Academic Hospitals and the accredited private units [47].

### Trauma governance, registry and performance improvement

There is no national governance. Trauma management has a multi-factorial governance supervised by the government with professional buy-in, and both private and public compliance and dual-payor system. There is no national trauma registry, while regional and facility-based registries exist in academic facilities and private groups. The Health Professions Council, Nursing Council, TSSA and College of Surgeons define quality assurance and trauma qualifications [47, 48].

### The South African trauma system facing major disasters and terrorist attacks

South Africa is no stranger to major incidents and disasters, with earthquakes, floods, and mass casualty incidents commonplace [49, 50]. During the Apartheid era, bomb explosions were commonplace and during the transition period around 1994 there were a number of grenade and gun-attacks on churches across the country [51, 52]. Due to the informal settlements with sheet-metal shacks built close together major fire disasters are commonplace. More recently there was extensive development in Disaster Medicine when the country hosted the football World Cup in 2010. This led to the national institution of the Major Incident Medical Management and Support (MIMMS) and Hospital Major Incident Medical Management and Support (HMIMMS) major incident response systems that have stood the country in good stead when planning and activating for the current SARS-CoV-2 pandemic [53, 54].

Daily there are mass casualty incidents when minibuses used as taxis with 15–20 passengers, buses or heavy vehicles crash with numerous casualties that overload the pre-hospital and hospital system. Train crashes occur at least one or twice a year, often with 150–200 casualties. Industrial and mining incidents are also noted about 2–3 times annually [55]. With the regular use of the MIMMS/HMIMMS approaches, despite stretched resources, the health system copes and comes out stronger on the other end.

### Current challenges for the South African trauma system

The challenge remains that many public hospital emergency departments are staffed by non-doctors, junior doctors and lack senior leadership, especially at district level where the focus is on primary care, disease prevention, and public health. This leads to the need for phased up-referral to regional and tertiary/quaternary facilities, delaying definitive care to the most vulnerable of the population. Lack of beds and access to ICU facilities, competition from other surgical cases, notably Caesarian Section, delay surgical care at most hospitals [39, 47]. The phased health care system “primary-care” model means that ambulances and private vehicles will often still take patients to the nearest facility, rather than the appropriate facility [39].

A vibrant and competitive private sector component has developed within the private health care arena. The challenge here is that the fee-for-service private sector cares for about 15% of the South African population yet uses up to 50% of the healthcare gross domestic product. With the exception of a small number of accredited Private level I and II trauma units, currently found only in Cape Town, Durban, Pietermaritzburg & Johannesburg, where dedicated trauma wards and a trauma ICU have been established, most private hospitals have no specific trauma wards and patients are accommodated in the ward of the specialist caring for them [46, 47]. Access to rehabilitation is remains limited in the public sector, while the private sector fairs much better on this aspect of trauma care [39, 56].

To date there is no national trauma databank or equivalent registry. There are facility-based registries and one large private hospital emergency registry, with disparate variables collected and different electronic platforms or even paper-based systems at the core [39]. There is no current national health identity system meaning that a patient record from one part of the country cannot be accessed elsewhere in an emergency, a major challenge to offering quality care to the injured.

Research on the trauma system in South Africa suggests improved return to work [39]. But it also faced with challenges regionally: the cost, infrastructure requirements and the risk of litigation, along with the reality of competing priorities, namely maternal-child-health, communicable disease (especially Tuberculosis and HIV-AIDS), and non-communicable lifestyle diseases in the context of limited gross domestic product to spend on health-care.

### Developing new trauma systems: requirements, consideration of specificities and difficulties

A trauma system consists of call and dispatch centers, pre-hospital care, in-hospital care, regional trauma networks, trauma registry, rehabilitation, and quality assurance [57]. Considering the pre-hospital care, several aspects are of critical importance: (1) the existence of a national or regional EMS with an access telephone number controlled by a lead agency; (2) the adaptation of EMS to the geographical characteristics and the population density; (3) the type of pre-hospital care: either physician- or paramedic-staffed. Physicians provide high quality ALS at the scene and during transportation to the hospital. Paramedics can deliver either ALS or basic life support (BLS) at the scene. ALS includes noninvasive and invasive interventions, such as endotracheal intubation and intravenous catheters for drug and fluid delivery (“stay and play”). BLS emphasizes rapid transport to the hospital with minimal treatment at the scene using only noninvasive interventions, such as bag valve masks for respiratory support (“scoop and run”); (4) the quality of triage and the type of transportation, either ground or air transportation, imply the existence of a regional trauma network to limit over and under triage and secondary inter-hospital transportations; (5) facility-based in-hospital care, assessed periodically by accreditation processes; (6) a national or regional trauma registry is critical for assessments and improvement. The construction of trauma systems in emerging countries is challenging. Any national trauma system is inevitably developed on the basis of the existing health-care system and infrastructures. China, Russia, and South Africa have experienced a difficult process, the primary trauma systems have already been established.

In China, due to the vast territory and an existing health-care system consisting of huge hospital resources with multidisciplinary capacities, the trauma system was conceived as regional, each region forming an inner trauma system as a part of the future national trauma system framework. High local population density, good economic conditions, dense ground transportation network, and the existing hospital resources were key determinants to select middle and eastern provinces as the first stage of the primary “CRTCS” (Fig. 1c). Each regional inner trauma system is centered on a large-scale level I trauma center, providing high quality in-hospital care, and meeting criteria of level IV hospital-based trauma system [57]. In the near future, aeromedical transportation will become a priority, to reduce the pre-hospital transportation time in the western provinces and border regions with difficult access terrain.

In Russia, the level III/IV pre-hospital trauma system [57] was formed within the past 20 years. Due to the vast territory, region-based coordination centers control the pre-hospital ambulance dispatch. The predominant distribution of trauma centers in the western and southern regions, fits the regional population density (Fig. 2). With a dense network of universities in these regions, a large number of emergency medical doctors are trained for delivering high quality pre-hospital physician-staffed ALS. Due to an outdated road network with a limited number of highways and railroads, and vast regions of difficult access, aeromedical care has been prioritized.

In South Africa, public and private health-care both contribute to province-based EMS but with no definite coordination protocol. The country has the best transportation network in Africa which contributes to facilitate ground EMS transportation. In-hospital care is inclusive and the number of accredited trauma centers is limited, resulting in most severely injured patients being transported to the nearest hospital. In-hospital care is managed by emergency medicine and multidisciplinary teams. Huge social disparities coming from the Apartheid period with persisting crowded townships in big cities, a vast territory with a relatively low density of population and lack of governmental priority for trauma care, explains why no unified trauma system currently exists in South Africa. An increase in financial support for trauma care, training in primary emergency trauma care, coordination and utilization of private health-care, also need to be planned.

## Conclusion

The construction of trauma systems in emerging countries is challenging, requires a thorough design and should be implemented according to a national plan. Flexible adjustments are adapted to specific country conditions. Trauma registry, often defective in the primary trauma system, is essential for the future developments and improvements in quality of care. Geographical, demographic and socio-economic conditions as well as communication network and financial input will markedly influence the development of trauma systems in emerging countries.

### Search strategy and selection criteria

References for this review were identified through searches of PubMed for articles published any date before February, 2021, by use of the terms “trauma system”, “trauma care”, “trauma center”, “pre-hospital care”, “in-hospital care”, “trauma registry”, “Chinese trauma system”, “Russian trauma system”, “South African trauma system”. Articles resulting from these searches and relevant references cited in those articles were reviewed. Articles published in English and Russian were included.

## Supplementary Information

**Additional file 1.** The Chinese trauma system: historical background.

**Additional file 2.** The Russian trauma system: historical background.

**Additional file 3.** The South African trauma system: historical background.

## Data Availability

All data generated or analyzed during this study are included in this published article [and its supplementary information files].

## Abbreviations

CTRTA: China Trauma Rescue & Treatment Association
NCTM: National Center for Trauma Medicine
CRTCS: Chinese Regional Trauma Care System
EMS: Emergency medical service
ALS: Advanced life support
TSSA: Trauma Society of South Africa
MIMMS: Major incident medical management and support
HMIMMS: Hospital major incident medical management and support
SARS-CoV-2: Severe acute respiratory syndrome coronavirus 2
HIV-AIDS: Human immunodeficiency virus-acquired immune deficiency syndrome
BLS: Basic life support
