# Supplementary material for: Management of severe trauma worldwide: implementation of trauma systems in emerging countries: China, Russia and South Africa
Source: Crit Care. 2021 Aug 9;25:286. doi: 10.1186/s13054-021-03681-8 (PMC8352140; doi:10.1186/s13054-021-03681-8)
Supplement: Supplementary file 1 — Additional file 1. The Chinese trauma system: historical background. [file 13054_2021_3681_MOESM1_ESM.pdf]

## The Chinese Trauma System: historical background

There were ancient records of traumatic disease treatment by traditional Chinese Medicine, including primary triage, emergency care on the scene, transportation, and military trauma care. Nevertheless, emergency rescue concerned mainly isolated trauma, with patients being managed by individual doctors, without any attempt at a trauma system. After the introduction of the western medicine into China, trauma care was handled by corresponding medical and surgical specialties. However, the pre-hospital emergency care was neglected, and trauma care was essentially depending on the in-hospital care, although some cities set up simple emergency sites. In the 1980s, the government held meetings, made policies for promoting the emergency care, and set the "120" as the national call number for emergency care. Subsequently emergency medicine was recognized and emergency centers were implemented in counties or cities to provide pre-hospital care.

In the last forty years, the in-hospital trauma care was mainly dispersed into individual specialties. Owing to the high incidence of limb trauma, the orthopedic surgeon was usually in charge of trauma care, assisted by other related specialties. After the emergency departments were created, they were in charge of trauma patient admissions, providing the initial care, and calling for consultation of other specialties according to the patient's injuries. In general, the most critical injury determined the specialty which was in charge of patient's management.

These conditions represented the usual mode of trauma care in China over the last forty years. Due to the deficiency of standard pre-hospital care and the lack of efficient communications between pre- and in-hospital phases, the quality of trauma care was unsatisfactory. Under the auspices of the National Ministry of Health, the Peking University Traffic Medicine Center

(PUTMC) conducted a national research project which revealed the deficiencies of trauma care in China, including long response time to manage an emergency call, long pre-hospital transportation time, long time for in-hospital care related to delayed arrival of specialists, deficiency of communication between pre- and in-hospital teams, deficiency of standard training for paramedics, too many specialties, deficiency of professional team for mass injuries and multiple injuries care, and deficiency in the standard protocols for the trauma care both on the scene and in-hospital. Solutions to solve these issues were suggested and a standard protocol for severe trauma care was established and extended nationwide. Many outcomes of trauma care were improved [1, 2]. These improvements were inspiring and created a new approach to trauma care in China with the understanding that one of the critical issues was the lack of a trauma system.

In 2015, a proposition for establishing the Chinese trauma system was revealed in the Lancet [1]. The idea was to create a system adapted to Chinese specificities, called the "Chinese Regional Trauma Care System". The aim was to establish regional hierarchical trauma system which includes professional paramedic-based teams for pre-hospital care, in-hospital emergency and multidisciplinary teams, pre-defined protocols for severe trauma care, integrative regional trauma databank, and educational training courses adapted to the specific situation in China. The first step was to select large-scale hospitals with multidisciplinary facilities to create the "Trauma Center" within the hospitals. The "Trauma Center" was connected to five to six small hospitals, called "Trauma Care Sites", to form a close-circle regional trauma system for triage, transportation and care. This design was summarized as "One region, Two links, Three teams". It took advantage of the existing numerous large-scale hospitals with multidisciplinary specialties, and avoided the difficulties and high cost that would have resulted from the creation of exclusive trauma centers [1,

3].

The China Trauma Rescue & Treatment Association (CTRTA), founded in 2016, was the first nationwide academic organization on trauma care in China. Under the promotion of CTRTA, the establishment of Chinese trauma systems spread nationwide. The "100 Counties Project" was a model of excellence which meant the founding of Chinese Regional Trauma Care Systems in at least 100 counties in China. In the last five years, this work gained rapid progress and resulted in better outcomes for trauma patients. The advancements of trauma care in China attracted the interest of international colleagues. In 2017 the Chinese scholars were invited to publish a review in the Lancet titled "Transport and public health in China" [4]. Currently "Chinese Regional Trauma Care System" has developed firm foundations, and has gained international recognition. The Kingdom of Cambodia and the Lao People's Democratic Republic have both signed memoranda with CTRTA to assist them in developing trauma systems in their countries.

**Abbreviations**

PUTMC: Peking University Traffic Medicine Center; CTRTA: China Trauma Rescue & Treatment Association

**Reference**

1. Wang TB, Yin XF, Zhang PX, Kou YH, Jiang BG. Road traffic injury and rescue system in China. Lancet 2015; 385(9978):1622.

2. Yin XF, Wang TB, Zhang PX, Kou YH, Zhang DY, Yu K, et al. Evaluation of the effects of standard rescue procedure on severe trauma treatment in china. Chin Med J (Engl). 2015; 128(10):1301-5.

3. Jiang BG. The challenges faced in the field of trauma care in China. Zhonghua Wai Ke Za

Zhi. 2015; 53(6):401-4. (in Chinese)

4. Jiang BG, Song L, Zhong-Ren P, Cong HZ, Morgan L, Qu C, et al. Transport and public health in China: the road to a healthy future. Lancet 2017; 390(10104): 1781-91.
