# Supplementary material for: Management of severe trauma worldwide: implementation of trauma systems in emerging countries: China, Russia and South Africa
Source: Crit Care. 2021 Aug 9;25:286. doi: 10.1186/s13054-021-03681-8 (PMC8352140; doi:10.1186/s13054-021-03681-8)
Supplement: Supplementary file 2 — Additional file 2. The Russian trauma system: historical background. [file 13054_2021_3681_MOESM2_ESM.pdf]

## The Russian Trauma System: historical background

The founder of modern Russian traumatology is Henry Turner (1858-1941), who organized the first clinic and the Department of Traumatology and Orthopedics in Russia on the basis of the Military Medical Academy in St. Petersburg in 1900. The second most significant figure is Roman Vreden (1867-1934), who founded the first traumatological institute in Russia in 1906 in St. Petersburg (today it is the Russian Scientific Research Institute of Traumatology and Orthopedics named after R. R. Vreden). However, conservative treatment methods dominated in the Russian traumatology in the first half of the 20$^{th}$ century, and the quantity of injuries that were treated operatively was negligible. This trend continued in the 60-80's of the last century. Despite the fact that harmonized methods of fracture surgical treatment were widely introduced in western European countries at that time, an extensive model of trauma care with a prevalence of conservative treatment methods and a lack of modern osteosynthesis and implant technologies was maintained in Russia [1].

Comparing the development of world and Russian traumatology in the second half of the 20$^{th}$ century, it has to be said that in the 60-70's of the last century, high-quality implants from various manufacturers of osteosynthesis were already created and presented on the world market as serial products [2]. The situation developed in a different way in the USSR (Union of Soviet Socialist Republics). In the country, internal fixation for fractures began to develop much later due to a number of historical and socio-economic reasons. The domestic medical industry produced only general surgical instruments, and the western countries markets were closed at that time. This fact predetermined the technological gap with developed countries of Europe and the United States. Due to the lack of serial implants production, representatives of almost every trauma school

offered their own variants of designs for osteosynthesis. They were often produced semi-artisanally, in small batches. Each Traumatology Department had its own locksmith's workshop for implant fitting to each specific patient. These circumstances were the cause of numerous complications of the surgical fracture treatment. However, by reviewing the failures and mistakes, the Russian trauma community has developed and moved forward. This time was dominated by external fixation methods offered by G. A. Ilizarov [3]. Nevertheless, the extensive but well-organized Soviet health care model worked quite well. All costs related to treatment were fully covered by the State.

In the early 90's of the last century there was a disintegration of the USSR. This process was accompanied by a systemic crisis and a global lack of financial resources, which resulted in the complete collapse of the Soviet health care system in general and the system of trauma care in particular. These processes could not but affect the social indicators.

By the beginning of the 21st century it became obvious that not only the political system had changed in Russia. Time had changed, the rhythm of life had become different. Against the background of the industry development and the appearance of numerous high-powered cars and trucks on the roads, the number of severe injuries increased. These factors helped to realize that the improvement of trauma care systems in Russia was necessary. The experiences of developed countries were studied. Many Russian trauma surgeons were given the opportunity to get fellowships in the best trauma clinics of Europe, which later allowed them to introduce modern injury treatment methods and organization of trauma care. The system of university and post-graduate education was also revised in the light of the latest achievements of medical science. Obviously, these changes required adequate funding and the State allocated a sufficient amount of

resources for the necessary equipment and staff training. As a result, a new system of trauma care, including pre-hospital care, in-hospital care and rehabilitation, was organized. A State program was adopted for reducing road traffic injuries, according to which 1500 trauma centers were organized throughout Russia. Level I trauma centers have well-equipped operating rooms, computed tomography and magnetic resonance imaging, ultrasound, angiography and have highly qualified staff to provide emergency assistance 24 hours a day [4]. The organized trauma care system reduced the amounts of injuries and the mortality of trauma.

**Abbreviations**

USSR: Union of Soviet Socialist Republics

**Reference**

1. Belenkiy IG, Khominets VV. Historical parallels in the development of the intramedullary osteosynthesis. State and prospects (literature review). Modern problems of science and education. Surgery.2020;5. doi:10.17513/spno.30055. http://www.science-education.ru/ru/article/view?id=30055 Accessed 08 Sep 2020.

2. Greenhagen RM, Jonson AR, Joseph A. Internal fixation: a historical review. Clin Podiatr Med Surg. 2011;28(4):607-18.

3. Tyulyaev NV, Vorontsova TN, Solomin LN, Skomoroshko PV. Development history and modern concern of problem of extremity injuries by external fixation (review). Traumatology and orthopedics of Russia 2011;(2):179-90. (in Russian)

4. Bugayev DA. Foreign and national experience in organizing tertiary medical care for injured in road accidents. Kazan Medical Journal 2019;100(3):464-8. (in Russian) doi: 10.17816/KMJ2019-464.
