# Supplementary material for: Management of severe trauma worldwide: implementation of trauma systems in emerging countries: China, Russia and South Africa
Source: Crit Care. 2021 Aug 9;25:286. doi: 10.1186/s13054-021-03681-8 (PMC8352140; doi:10.1186/s13054-021-03681-8)
Supplement: Supplementary file 3 — Additional file 3. The South African trauma system: historical background. [file 13054_2021_3681_MOESM3_ESM.pdf]

The South African Trauma System: historical background

Most of Africa has an underdeveloped, or in many places a non-existent trauma system, with sporadic exceptions. There are, however, many centers of excellence in a sea of indifference to the plight of the injured, due to the myriad of competing health priorities. Therefore, the system has to be afrocentric in nature, addressing the disparities of the people [1, 2]. The global trauma burden is heavily weighted toward Africa, with almost 90% of the trauma deaths in Africa and the near East, especially in Lower-Middle-Income-Countries (LMICs), yet with the least developed systems [3]. The evolution of the South African trauma system to where it is currently has progressed from a completely non-emergency oriented communicable disease/non-communicable disease hospital-centric system to a system with defined levels of care, national core standards for care and quality assurance guidelines implemented through the national health department, applicable to public and private healthcare facilities alike [1, 2].

South Africa preceded the USA by some years in the establishment of a designated trauma facility. The first trauma “center”, called the Accident Service, was established in 1962 at the Johannesburg Hospital by Dr Wilkinson, after his visit to Birmingham in 1960, and was supported by the Medical School of the University of Witwatersrand [2]. However, until the early 1980’s there were no defined trauma systems in any African countries, with even pre-hospital emergency care in its infancy. Ambulances had minimal equipment, except for South Africa, and required little more than a driver and no formal training. South Africa at least required a basic first-aid certificate and most vehicles had two staff, a driver and an attendant [4]. In the 1980’s the concepts of emergency paramedic care started to filter across the Atlantic from the USA and the UK. In Cape Town and KwaZulu-Natal, emergency services employed doctors to set up paramedic programs

and the first real Emergency Medical Service (EMS) came into being. Dr's Alan McMahon in Cape Town and Alan White along with John Keenan in KwaZulu-Natal initiated the first paramedic programs in the country [5]. This led to the development of specialized medical rescue services across the country either within the Ambulance service or as part of the Fire Service.

The first post-graduate medical qualification in emergency care was developed in 1986 by C. van der Merwe, M. Morris and J. White, with the establishment of the Diploma in Primary Emergency Care (initially as part of the Faculty of Family Medicine, currently resorting under the College of Emergency Medicine) as a part-time higher training in emergency care for non-specialists. This diploma has proven popular with general practitioners working in pre-hospital care and also casualty officers and remains so to the present [1]. Hospital programs followed with Cape Town facilities (Groote Schuur and Tygerberg Hospitals) among the first "designated" trauma center in the southern hemisphere, following from the Johannesburg example. Subsequently trauma care developed as part of the general surgical services at many other regional and teaching facilities, either as undifferentiated general surgical emergency services or as defined trauma units such as the one at Inkosi Albert Luthuli Central Hospital (IALCH) in Durban, the first Trauma Society of South Africa (TSSA) accredited public level I trauma center in the country [1, 2]. The TSSA was founded in 1983 to gather like-minded professionals of all disciplines under one umbrella organization. Members include doctors, nurses, allied health professionals, pre-hospital practitioners and intensivists, all with the desire to improve the care of the injured. The TSSA became the custodian of the Advanced Trauma Life Support (ATLS™) program in South Africa in 1992 and the Definitive Surgical Trauma Care course (DSTC) from 1999. It implemented a set of trauma-center accreditation guidelines published in 2011, currently in a revision and update phase.

Additionally, with the support of the TSSA, Trauma Surgery became the 4th recognized surgical sub-specialty in South Africa, with a post-surgical two-year “certificate” program in trauma and critical care (open to general surgeons after completion of the Fellowship Exam). Research programs and teaching programs at local universities have evolved emphasize to students and registrars the essential theoretical and practical aspects of trauma care, given the high trauma burden in the country. Much of the development has been spurned by the Essential Trauma care and Pre-hospital Trauma Care programs of the World Health Organization (WHO) [6, 7] and more recently through the African Federation of Emergency Medicine, the Trauma Society of South Africa and similar professional societies [1].

In terms of pre-hospital care, the progress has been even more dramatic, with initially basic and later short-course based advanced paramedic programs in the 1980’s and 1990’s being replaced by initially the three-year diploma and more recently four-year degree paramedic programs. They now have their own independent professional board, nationally regulated scopes of practice and evidence-informed practice guidelines [8, 9]. Since this is nationally standardized, there is some coherence in the pre-hospital system, although many practical aspects must be expanded, such as inter-service communication and cooperation, particularly among the private services, and a move away from taking patients to the nearest facility, rather to the most appropriate facility. Quality assurance programs and clinical audit of paramedic treatment is developing at a reasonable pace [10]. Masters and PhD programs exist at the various Universities of Technology.

In terms of intra-hospital care, treatment of trauma improved through the implementation of emergency medicine programs in 2004 and the training of emergency medicine specialists in 2007. This has moved the care of emergencies from the realm of the “interested amateur” to

consultant-led services at a number of the major referral centers around the country. This has led to improvements in trauma initial care. Some hurdles are still present, such as timely involvement of the surgical team, or alternatively the lack of an available surgical team as they are engaged in the operating room. Access to sufficient emergency blood, the development of cheap autotransfusion devices [11] and surgical skill training of the emergency specialists to perform certain life-saving procedures has reduced the mortality in certain areas. Definitive surgical and intensive care for trauma has also shown progress since the 1990's: the first academic units for trauma were established in the early 1990's and trauma care was recognized as an academic discipline by many surgical departments. With the development of the concepts of intensive care in the 1980's and damage control in the late 1990's it became clear that trauma surgery was not the same as general surgery, the former focusing on physiology more than anatomy. This led to the College of Surgeons requiring a defined training time in trauma for all surgical disciplines (between three and nine months, depending on the specialty) and the eventual development of the post Fellowship of the College of Surgeon Certificate in Trauma, a two-year program of 12 months exposure in trauma operative and trauma intensive care respectively [12]. These trauma surgeons now provide the leadership and future development of the system through both the public and private sectors. Trauma nursing care has developed through the recognition of emergency nursing and intensive care nursing as specialist nursing qualifications in South Africa and there is ongoing demand for training [13]. Allied health professionals, particularly physiotherapy and occupational therapy have a major role in the restoration of health after trauma. While the role of these and other disciplines (dietetics, speech/audio therapy and psychology) are essential in the care of the trauma patient it is only recently that advanced training in either trauma or ICU aspects has developed for most of

these disciplines.

Abbreviations

LMICs: Lower Middle Income Countries; EMS: Emergency Medical Service; IALCH: Inkosi Albert Luthuli Central Hospital; TSSA: Trauma Society of South Africa; ATLS: Advanced Trauma Life Support; DSTC: Definitive Surgical Trauma Care; WHO: World Health Organization

Reference

1. Hardcastle, TC. A Trauma System for KwaZulu Natal-Local Development for Local Need. PhD Thesis 2013, University of KwaZulu-Natal. https://researchspace.ukzn.ac.za/xmlui/bitstream/handle/10413/11582/Hardcastle_Timothy_Craig_2013.pdf?isAllowed=y&sequence=1. Accessed on 01 Jan 2014

2. Hardcastle TC, Brysiewicz P.Trauma in South Africa: From humble beginnings to an Afrocentric outreach. Int Emerg Nurs. 2013; 21:118-22.

3. Peden M, Hyder AA. Time to keep African kids safe. S Afr Med J.2009;99:36-7.

4. MacMahon AG. The mobile medical squad. S Afr Med J.1974;48:1915-9.

5. MacMahon AG. Incidents and Accidents: Metro Rescue the Early Days. Self-published, 2018 ISBN 9780620659598.

6. Mock C, Lormand JD, Goosen J, Joshipura M, Peden M. Guidelines for essential trauma care. Geneva,World Health Organization 2004.

7. Sasser S,Varghese M, Kellermann A, Lormand JD. Pre-hospital trauma care systems. Geneva, World Health Organization 2005.

8. Health Professions Council of South Africa, Professional Board for Emergency Care: Clinical Practice Guidelines for Emergency Care, July 2018, HPCSA, Pretoria ZA.

9. Emergency medical services regulations. Government Gazette No 37869, 24 July 2014, Department of Health-South Africa.

10. Muhlbauer D, Naidoo R, Hardcastle T. The History of Helicopter Emergency Medical Services within South Africa. Presented at the Aeromed Africa Conference, 2012.

https://silo.tips/download/history-of-helicopter-emergency-medical-services-in-south-africa Accessed on 20 Aug 2020.

11. Hardcastle TC. Cheap, but efficient trauma care in South Africa: Sinapi® autotransfusion-Plenary Lecture at SWAN-21, Sydney Australia, 23 July 2013.

12. College of Surgeons of SA. Regulations for the Sub-speciality certificate in Trauma Surgery. Accessed from https://www.cmsa.co.za/view_exam.aspx?QualificationID=103 on 23 Sep 2019.

13. Brysiewicz P, Bruce J. Emergency nursing in South Africa. International Emergency Nursing 2008;16:127-31.
